# Association between Oesophageal Diverticula and Leiomyomas: A Report of Two Cases

**DOI:** 10.1155/2016/6832535

**Published:** 2016-11-03

**Authors:** Muhammad Chowdhry, Christina Spyratou, Bruno Lorenzi, Sritharan Kadirkamanathan, Alexandros Charalabopoulos

**Affiliations:** Department of Upper Gastrointestinal Surgery, Broomfield Hospital, Mid Essex Hospital Services NHS Trust, Essex, UK

## Abstract

We report two rare cases of female patients presenting with oesophageal leiomyoma associated with oesophageal diverticulum, both of whom were surgically managed. Oesophageal leiomyoma and oesophageal diverticulum are uncommon as separate entities and rare as combined disease presentation. Clinicians need to be aware of the rare combination of the two entities and need to be able to exclude the presence of a tumour (benign or malignant) within a diverticulum and so plan the optimum treatment. Herein, we present two cases of oesophageal leiomyoma within oesophageal diverticulum and we try to elucidate the association between the two. To date, there is no consensus whether a diverticulum is secondary to a leiomyoma or, on the contrary, a leiomyoma arises within a diverticulum.

## 1. Introduction

Oesophageal diverticula and oesophageal leiomyomas are infrequent benign conditions of the oesophagus that may render a potentially difficult diagnosis as well as challenging management. Simultaneous existence is even scarcer and there are only a limited number of cases reported in the English published literature [1]. In terms of the pathogenesis of the two synchronous entities, to date it is still unclear which of the two is preexisting and which results in the presence of the first one. Different schools of thought are in support of each theory (diverticulum leading to leiomyoma or leiomyoma to diverticulum), without a firm favorite so far. Their management varies from conservative to local excision for diverticula and from enucleation to oesophagectomy for leiomyomas [2, 3]. The diagnosis of the two entities preoperatively can be difficult to establish and frequently the discovery of the leiomyoma within the diverticulum is an incidental finding. Clinicians involved in the diagnosis and management of oesophageal conditions need to also bear in mind that there is a low probability of cancerous transformation within the diverticulum [4]. This needs careful consideration regarding best investigations and management of these patients. Most oesophageal conditions, including diverticula and leiomyomas, are nowadays being dealt with in high volume tertiary centers where great expertise and multidisciplinary team effort have provided better results in the management and outcome of these indeed challenging conditions [2]. Therefore, careful history, investigations, and clinician's awareness towards possible synchronous disease is imperative in order to ensure the best outcome for these patients. In the current study we describe two interesting cases of synchronous disease as well as their management and follow-up. Furthermore, alongside a literature review, we provide a description of the two disease entities and we try to elucidate their combination, which complementary could help immensely any clinician dealing with oesophageal conditions.

## 2. Case Report 1

**Figure 1:**
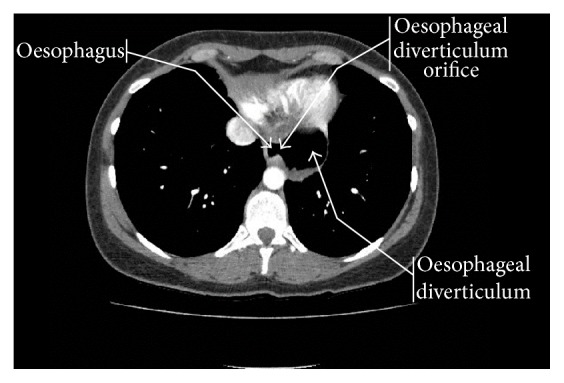
Epiphrenic oesophageal diverticulum containing food residue. CT chest with IV contrast.

**Figure 2:**
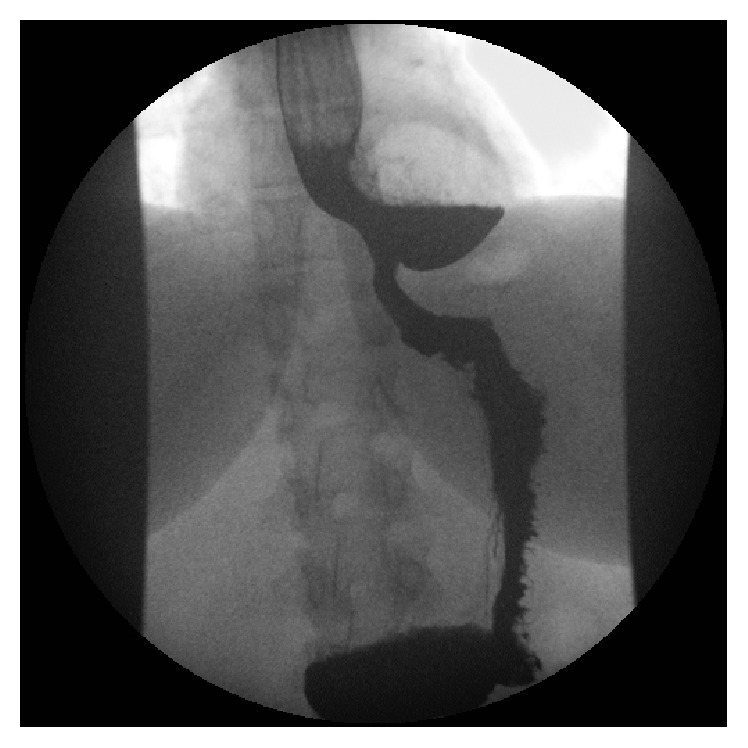
Oesophageal diverticulum with air-fluid level. Barium swallow.

A 35-year-old woman presented to the upper gastrointestinal clinic with dysphagia, odynophagia, reflux, and persistent bloating after meals. Her abdominal ultrasound did not detect any abnormalities. Subsequent oesophagogastroduodenoscopy (OGD) revealed the presence of a paraoesophageal hernia within which two 4 cm cream lesions were apparent. Biopsies of the area showed mild chronic inflammation and intestinal metaplasia consistent with Barrett's oesophagus and no dysplasia. A CT scan revealed that what was thought to be a paraoesophageal hernia in the OGD was in reality a 5.5 × 4.7 cm epiphrenic oesophageal diverticulum containing food residue (Figure 1). Finally, barium swallow revealed gross reflux up to the upper third of the oesophagus and a large distal oesophageal diverticulum (Figure 2).

Due to the severity of symptoms, surgical intervention was planned. Intraoperative findings confirmed an oesophageal diverticulum near the gastrooesophageal junction. Robotic-assisted laparoscopic excision of the epiphrenic diverticulum with distal oesophageal myotomy and hiatal closure with crural repair was performed. Postoperative water-soluble swallow demonstrated no leak from the diverticulectomy site and some narrowing at the gastrooesophageal junction likely due to postoperative oedema. The patient had an uneventful recovery and was discharged home two days later. Histology showed an oesophageal diverticulum of 55 × 35 × 20 mm containing a firm lesion of 30 mm. The diverticulum was lined by gastrooesophageal mucosa and the wall contained a lobulated smooth muscle tumour arising from the muscularis propria. The lesion composed of spindle cells with no evidence of nuclear pleomorphism, necrosis, or increased mitotic figures. Immunohistochemistry found lesion cells positive for SMA and desmin and negative for CD34, CD117, DOG1, and S100 protein and concluding that the appearance was in keeping with leiomyoma with no evidence of malignancy. A year later, a follow-up CT was performed, with no abnormalities detected.

Two years later, she complained of increasing reflux symptoms and mild dysphagia especially after large meals. Barium swallow showed free flow of contrast into stomach. Further filling the stomach with a larger quantity of barium and tilting the fluoroscopy table to a semisupine position demonstrated gross gastrooesophageal reflux into a dilated oesophagus. A year later, she underwent laparoscopic 270-degree posterior fundoplication (modified Toupet) to control her reflux symptoms, with good results to date.

## 3. Case Repot 2

**Figure 3:**
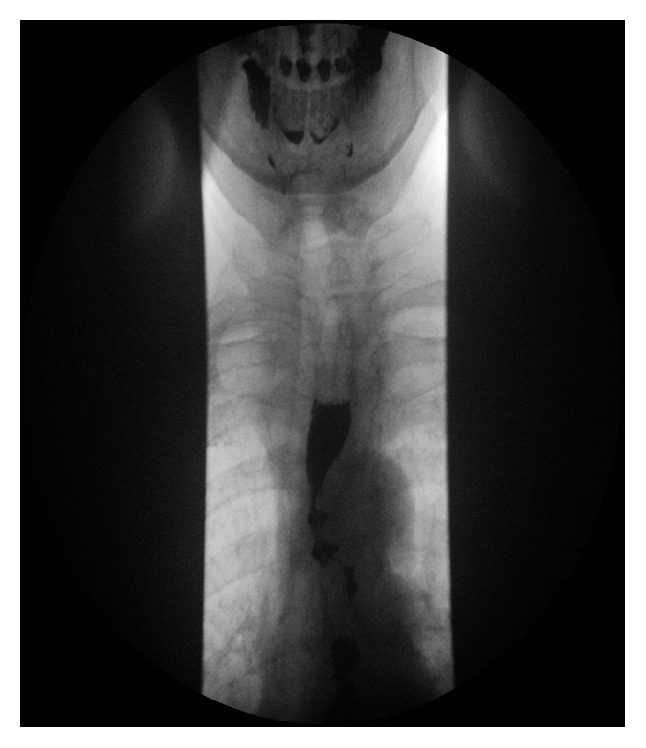
Corkscrew oesophagus. Barium swallow.

A 72-year-old woman presented with a 20-year history of dysphagia. She complained of heartburn but no weight loss. She had a barium swallow, which revealed corkscrew oesophagus (Figure 3). Her symptoms were improving with GTN spray.

Station pull-through manometry revealed grossly abnormal oesophageal body motility with very hypertonic contractions in the mid and distal body and concluded on diffuse oesophageal spasm involving at least 15 cm of the distal oesophagus.

She had an OGD where marked tertiary oesophageal contractions were present and the stomach could not be visualized, as it could not be intubated.

After failure of Botox injections (300 Units) to control her symptoms, she underwent a laparoscopic-assisted oesophagogastrectomy for corkscrew oesophagus.

**Figure 4:**
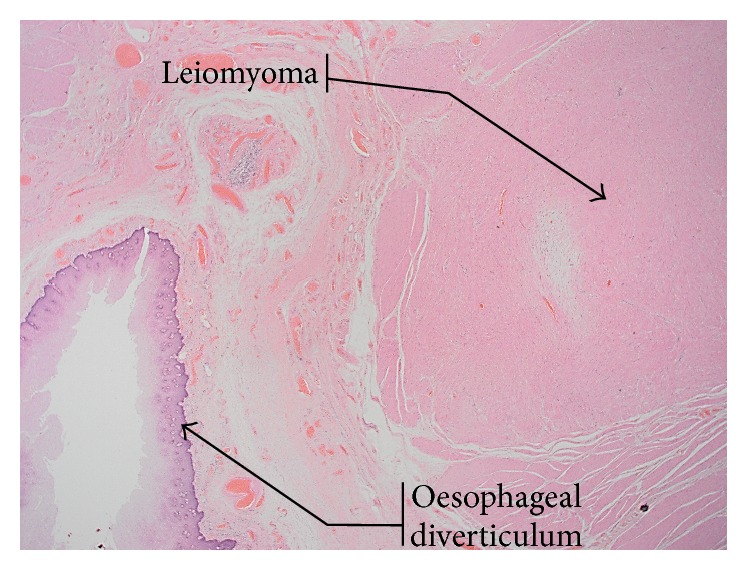
Histological view showing leiomyoma and oesophageal diverticulum. Haematoxylin & eosin stain ×10.

Histology revealed an oesophageal diverticulum 44 mm from the proximal oesophageal resection margin and 27 mm from the gastrooesophageal junction (GOJ) containing small amount of food debris. Also a smaller diverticulum was present nearby lined with squamous mucosa. The larger diverticulum contained a small fibrous nodule of 9 mm, in the outer layer of the muscularis propria at the proximal end of the diverticulum. It was identified as a benign leiomyoma (Figure 4). Immunohistochemistry was positive for SMA and desmin and negative for CD34 and CD117 and DOG.

She recovered well from the operation, but over the next few months she progressively developed symptoms of delayed gastric emptying. An upper gastrointestinal endoscopy was performed that showed a patent anastomosis and increased food residue within the gastric conduit. A barium swallow showed no anastomotic stricture but hold-up of contrast at the pylorus. Following the OGD, the patient complained of progressive and severe vomiting triggered by meals until not being able to tolerate solids, fluids, or medication.

She was diagnosed with pylorospasm after oesophagectomy (secondary to vagus nerve injury) and underwent endoscopic treatment. She had endoscopic balloon dilation and Botox injection of the pylorus to improve gastric drainage, which relieved her symptoms to date.

## 4. Discussion

Oesophageal diverticulum is a rare condition that can be managed conservatively when asymptomatic or surgically when symptoms become debilitating. An oesophageal diverticulum is defined as the outpouching or sac of the epithelial lined tissue of the oesophagus. True diverticula involve all the layers of the oesophagus, whereas false diverticula are limited to involving the mucosal or submucosal layers protruding through the circular and longitudinal oesophageal muscle layers [5].

Oesophageal diverticula can be classified by location of occurrence, be it proximally at the pharyngooesophageal area (Zenker diverticulum), the mid oesophagus, or more distal (epiphrenic) diverticula [2]. The mechanism of occurrence is thought to be either traction (due to scaring and conditions following chronic inflammation such as TB) or pulsion (due to motility disorders of the oesophagus such as achalasia) and diverticula can be named accordingly. Rarer causes include retained foreign bodies [6, 7] and iatrogenic causes (oesophageal stents [8], gastric bands [9], and tracheostomy tubes [10]). Malignant lesions such as squamous cell carcinoma have also been described as possible cause of oesophageal diverticula [11, 12].

Oesophageal leiomyomas on the other hand are rare accounting for about 1% of all oesophageal neoplasms; however, they are the most common benign tumours of the oesophagus [13]. It is more common in men and usually arises from the inner circular muscle layer at the distal oesophagus. They are similar to classic endometrial leiomyoma as originally described consisting of a circumscribed lesion of muscularis propria or muscularis mucosae composed of intersecting fascicles of bland spindle cells with abundant cytoplasm and may show calcification. They stain positive for desmin, SMA (alpha smooth muscle actin), and high molecular weight caldesmon and negative for CD34, CD117, S100, and DOG1/ANO1. Oesophageal leiomyomas are typically treated with enucleation (open or thoracoscopic) for small tumours and oesophagectomy for large tumours [14, 15].

Oesophageal diverticula associated with leiomyomas although considered to be rare are reported in the literature since 1958 [16]. Several cases have been described since then but the etiology has not been clear yet. It is possible that the mechanism of diverticulum formation involves pulsion due to motility disorders caused by the leiomyoma itself (especially if it is bulky). In most of the cases the diverticula associated with leiomyoma are in the lower part of oesophagus (epiphrenic), which are typically considered to be pulsion diverticula. In our series, both cases had oesophageal motility disorder. However, recently a case of epiphrenic diverticulum associated with leiomyoma in a patient with no primary motor disorder has been described [1]. Therefore, other causes like traction due to surrounding tissue inflammation must be considered as well. Furthermore, for reasons that are not very well understood, there is a thought that leiomyomas can develop within a primary oesophageal diverticulum.

The incidence of cancer in a diverticulum is 0.3–7%, 1.8%, and 0.6% for pharyngooesophageal, midoesophageal, and epiphrenic diverticula, respectively [4]. Benign tumours are even rarer. Malignant transformation in a diverticulum is linked to stasis and it should always be taken into consideration when treating a long-standing oesophageal diverticulum.

In our series both patients had epiphrenic diverticula associated with leiomyomas. Both patients had oesophageal motility disorders and leiomyomas were only discovered histopathologically in the resected specimen. In both cases it is unclear if the diverticulum preexisted the leiomyoma or vice versa; however in our opinion given the findings we conclude that most probably these represent pulsion diverticula.

To date, in most of the cases reported, it is not possible to tell which comes first (the diverticulum or the leiomyoma). More studies are needed so as to be able to identify the primary cause in this combined disease.
